# Achieving precision surgery in laparoscopic liver resection with the aid of preoperative three-dimensional reconstruction: A case report

**DOI:** 10.1016/j.ijscr.2021.105792

**Published:** 2021-03-18

**Authors:** Filippo Banchini, Enrico Luzietti, Sara Cecconi, Marta Ribolla, Gerardo Palmieri, Patrizio Capelli

**Affiliations:** a Department of General Surgery, Guglielmo da Saliceto Hospital, Piacenza, Italy; b Department of Surgery at Università degli Studi di Parma, Italy

**Keywords:** Three-dimensional, 3D, Liver, Metastases, Laparoscopy, Hepatectomy, Case report

## Abstract

**Introduction:**

The use of three-dimensional image reconstruction in liver surgery is well-known and has got many applications: It was first developed for vein reconstruction in liver transplantation and for liver volumetry to prevent post hepatectomy liver failure (PHLF) after major resections. There are many other advantages described in the literature provided by three-dimensional reconstruction, however its diffusion is currently limited.

**Clinical case:**

We present the case of a woman with a single colon cancer metastasis in segment 5 of the liver. Using CT scan images we created a three dimensional reconstruction of the patient’s liver and its inners structures. The rendering was used to hypothesize the plan of dissection and to predict the pedicles that needed to be dissected during the procedure.

**Discussion:**

We try to demonstrate that, thanks to three dimensional image reconstruction, all the structures that need to be dissected could be effectively located prior to the the surgery with a high grade of approximation. Furthermore the 3D reconstruction could be used as a step by step guide during the whole surgical procedure, showing all the pedicles To be encountered and dissected at every stage.

**Conclusion:**

3d reconstruction of the liver is a valid aid in the interpretation of preoperative imaging and intraoperative ultrasound, both for the surgeon and for the entire equipe, facilitating comprehension of patient’s liver anatomical features. It allows to predict the location and direction of the pedicles that need to be dissected and resected with high approximation, in order to achieve a more precise and tailored surgery.

## Highlights

• Laparoscopic liver surgery is evolving and its spread is now starting to take place.
• Three-dimensional reconstruction imaging still remains a scarcely used technique.
• Considering the difficulty of liver resection surgery, a precise knowledge of the patient’s anatomy is essential in the preoperative planning of the intervention. Three-dimensional reconstruction imaging provides useful information in addition to conventional imaging, allowing a more accurate preoperative planning, and being used as a navigation instrument during liver resection.
• The use of three-dimensional reconstruction imaging allows to predict the precise location and direction of anatomical structures with high approximation, allowing to reach a high degree of precision surgery.

## 1 Introduction

Surgical treatment strategies of liver lesions is based on preoperative CT and MRI imaging studies. In almost all cases, treatment options and surgical strategy are assessed from the evaluation of two-dimensional imaging. The first application of 3D reconstruction imaging reported in literature aimed to assess the extent of disease in intrahepatic cholangiocarcinoma [1]. Imaging-based liver volumetry was developed in an attempt to avoid complications from insufficient liver remnant after major resections (post hepatectomy liver failure) [2]. Other authors proposed 3D living donor CT hepatic venography in liver transplantation, to prevent transplanted organ failure due to vein congestion [3]. Moreover subsequent studies have confirmed that image extraction from CT and MRI allows for remarkably useful three-dimensional reconstructions of the patient’s liver, that can be used for direct visualization and study of key structures on the screen and furthermore to obtain a 3D printed model of the organ. Many advantages of this technique are described in the literature [4], but its diffusion is currently limited due to its presumed high costs and need for sophisticated softwares. As a matter of fact there are many softwares available, some of which are completely free. In this clinical case, managed at our Institute, we try to demonstrate how both the planning of the procedures and the feasibility of interpretation of 3D images allow for performing surgery with an high degree of precision.

### 1.1 Clinical case description

We describe the case of a 53 Y/o woman with no previous clinical and drug history, hospitalized for occlusive left sided colon cancer with synchronous single 4.7 cm liver metastasis in segment 5, and treated with urgent left hemicolectomy and subsequent colostomy for anastomosis leakage. In consequence of the long postoperative period, in order to reassess the burden of the disease the patient underwent a new abdominal CT scan, which showed an unexpected reduction in size of the liver metastasis. A multidisciplinary discussion was conducted, resulting in the indication for liver resection. An anathomycal segmentectomy 5 was planned.

## 2 Material and methods

**Fig. 1:**
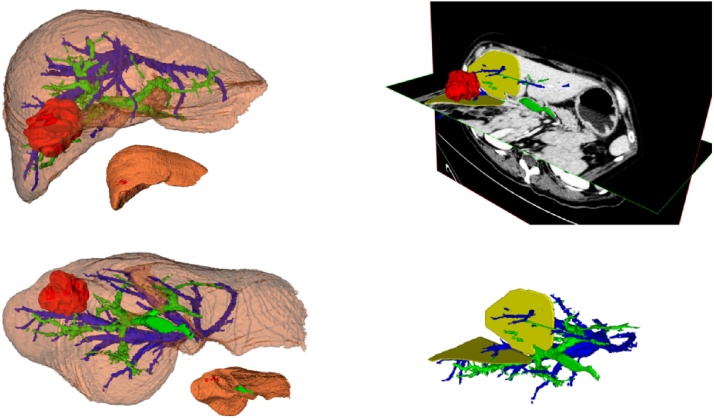
Preoperative liver rendering: Liver reconstruction with layers’ transparency and dissection plans hypothesized: in Red liver metastasis; in Blu hepatic veins; in Green Portal Branches; in Yellow hypothesized plans of dissection.

Portal phase Dicom data were uploaded in INvesalius® software. Separate layers for liver, portal branches, hepatic veins and liver metastasis were created. In this software each layer has got the capability of transparency regulation to better visualize every single structure. Once the three-dimensional reconstruction had been done, we used the model to precisely locate the tumor and estimate their relations with the portal and hepatic branches. We then planned the procedure by hypothesizing the liver's dissection plan, and predicting all the vascular structures crossing it (Fig. 1).

During the planning of the intervention, we recognized four main pedicles crossing the plan between segments 5 and 8 and two portal pedicles for segment 5 originating from the right anterolateral branch. Along the plan of the right hepatic vein, we identified four veins arising from it and a small portal pedicle from the branch for segment 6.

## 3 Description

Both 3D reconstruction and laparoscopic surgical procedure were lead in November 2020 by the first author, an experienced laparoscopic and liver surgeon, assisted by two more operators.

The intervention was conducted with patient in left lateral decubitus position. Trocars were placed at the margin of the right upper quadrant in a semicircular arrangement.

During the laparoscopic intervention, intraoperative ultrasound was performed, confirming the location and depth of the metastasis and guiding the marking of the dissection plan on the liver surface.

We started the dissection from the pericholecystic plan, at the apex of segment 4. Three-dimensional images were made available on screen inside the operating theatre; as the dissection proceded these images could be consulted in real time, guiding the step by step search for all the pedicles that had already been identified in the preoperative planning phase.

**Fig. 2:**
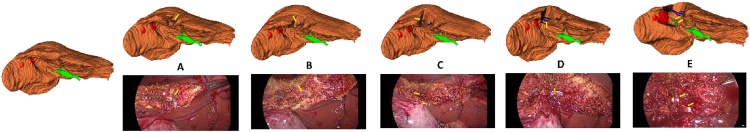
Pedicles dissected during hepatotomy between segment 5 and 8. Yellow arrows indicate the corresponding pedicle during the procedure; in Blu hepatic veins; in Green Portal Branches; in Yellow corresponding pedicle dissected.

In the separation plan between segments 8 and 5, we found four principal pedicles that were recognized, clipped, and sectioned. Once we had opened this plan, the dissection continued with the search for the right anterior portal branch, where we found two main pedicles for segment 5, as predicted (Fig. 2).

**Fig. 3:**
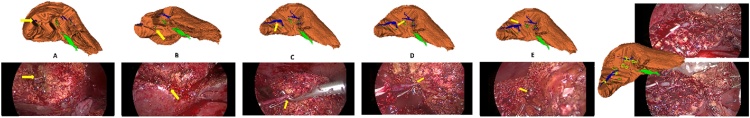
Pedicle dissected during hepatotomy along the plan of right hepatic vein. Yellow arrows indicate the corresponding pedicle during the procedure; in Blu hepatic veins; in Green Portal Branches; in Yellow corresponding pedicle dissected.

The following dissection started from the apex of segment 6 using the right hepatic vein as a marker. As already predicted during the three-dimensional reconstruction, on this plane we found four veins tributaries of the right hepatic vein and one portal pedicle originating from the branch for segment 6 (Fig. 3). All these structures were identified, dissected, and resected as showed in the video.

We achieved a precise anathomycal resection of segment 5.

Post-operative recovery course was uneventful, except for mild fever, which was treated with empirical antibiotic therapy (Score I, according to Clavien-Dindo Classification) and was discharged on postoperative day 8. At present, the patient is under adjuvant FOLFOX chemotherapy, in good general conditions.

## 4 Discussion

The use of three-dimensional reconstruction in liver surgery is well-known. Its applications range from vein reconstruction in liver transplantation [3] to organ volumetry [1] to prevent post hepatectomy liver failure after major resections.

The availability of dedicated rendering softwares have added further advantages, enabling intra-organ visualization and precise reconstruction of the patient’s liver different features and vascular structures. Many authors [5,6] have proposed the 3d printing of the reconstructed organ for a better understanding of the patient’s anatomy, but this technology can be expensive and time consuming. In our opinion, the real advantage of 3d software comes from two particular characteristics: the first is the possibility of adjustable transparency of the multiple layers of the 3D model, each representing a different feature, making it possible to highlight the structure of interest (i.e. showing a 3D picture of the portal vascular system branching and its relations to the tumor mass by making the surrounding parenchyma invisible) and the second is the possibility to rotate the 3D model in every direction, simulating the organ's manipulation in one’s own hands. In this way, we can visualize the organ from every angle, creating plans of visualization and hypothesizing plans of dissection that are impossible to conceive with the classical 2d reconstruction of a CT scan. The result of this imaging processing is so realistic and interactive that, in addiction to be used for accurate preoperative planning, it could also be consulted during the intervention as a navigation tool to predict all the structure that are going to be encountered, dissected and resected along the dissection plan, as we already reported in one of our previous presentations [7]. Therefore, this technology can be used in combination with intraoperative ultrasound, that remains a fundamental tool for the liver surgeon, as it can help in the interpretation of intraoperative ultrasound real time findings, while on the other hand ultrasound scanning of the liver can be useful in verifying the exact position of intraparenchymal structures previously visualized in 3d reconstruction. This imaging cooperation modality allows for the right choice of the dissection plan and its direction through the liver parenchyma until finding the prefixed landmarks visualized in the 3d reconstruction.

As demonstrated in our clinical case, and as previously reported by other authors [8], this hybrid strategy allows for punctual intraoperative navigation and a high grade precision surgery.

Using this methodology, instead of opening the dissection plan and finding vascular structures, we were able to direct the dissection process towards the way determined by the preliminary search for vascular pedicles.

## 5 Conclusion

Three-dimensional reconstruction imaging can be considered a powerful tool in liver resection, allowing to visualize the organ and its inner structures from every possible angle, which is impossible to achieve with standard 2d imaging. This technique is a valid aid in the interpretation of intraoperative ultrasound both for the surgeon and the entire equipe, facilitating anatomical comprehension. It allows to predict with high approximation the pedicles that need to be dissected and resected, in order to achieve a more precise and tailored surgery.

The case report follows both the SCARE and PROCESS Guidelines [[9], [10], [11]].

## Declaration of Competing Interest

The authors report no declarations of interest.

## Sources of funding

No found sources.

## Ethical approval

No ethical approval is require, because it is a single case report and the patient signed consent form for publication.

## Consent

Written informed consent was obtained from the patient for publication of this case report and accompanying images. All reported images and discussion protect anonymity.

## Author contribution

Banchini Filippo: Conceptualization, Methodology, Software, Resources, Writing - Original Draft, Writing - Review & Editing, Visualization, Supervision, Project administration.

Luzietti Enrico: Writing - Review & Editing.

Cecconi Sara: Writing - Review & Editing.

Ribolla Marta: Writing - Review & Editing.

Palmieri Gerardo: Writing - Review & Editing.

Capelli Patrizio: Writing - Review & Editing.

## Registration of research studies

Not applicable.

## Guarantor

Filippo Banchini.

## Provenance and peer review

Not commissioned, externally peer-reviewed.
